# Evaluation of the Symptom-Based Diagnostic Criteria for Irritable Bowel Syndrome in Conjunction With Clinical Examinations and Laboratory Investigations

**DOI:** 10.7759/cureus.38567

**Published:** 2023-05-05

**Authors:** Shaurav Ghosh, J V Pranav Sharma

**Affiliations:** 1 General Surgery, East Point College of Medical Sciences and Research Centre, Bengaluru, IND; 2 General Surgery, Adesh Medical College and Hospital, Kurukshetra, IND

**Keywords:** functional/diagnosis, abdomen pain, irritable bowel syndrome, surveys and questionnaires, colonic diseases

## Abstract

Background

Irritable bowel syndrome (IBS) is a chronic condition characterized by persistent abdominal pain or discomfort and impaired bowel function. Symptoms often vary in onset and severity, are worse during flare-ups, and affect the patient's quality of life. A positive diagnosis of IBS based on clinical symptoms may lead to a better outcome. There are different diagnostic criteria like Kruis score, Manning criteria, Rome I, II, III, and IV criteria, and each new one addresses the deficiencies of the previous ones. We analyze the effectiveness of the most commonly used diagnostic criteria associated with clinical examinations and laboratory tests in treating IBS in these studies.

Methodology

This is a retrospective study in which data from IBS subjects were collected by simple random sampling and compared using Manning criteria, Kruis score, and Rome IV criteria. Laboratory tests included complete blood count (CBC), erythrocyte sedimentation rate (ESR), and C-reactive protein (CRP).

Results

Of the 130 patients, IBS is more prevalent in adults aged 30-50 years, with a male predominance. The Kruis score outperformed the Manning criterion in distinguishing between organic bowel disease and IBS. This, together with the Rome IV criteria, increases the likelihood of identifying IBS.

Conclusions

Differentiating IBS from functional and organic gastrointestinal problems is critical. Irritable bowel syndrome can be diagnosed using symptom-based diagnostic criteria. Clinical observation and physical examination should be supplemented with laboratory indicators.

## Introduction

Irritable bowel syndrome (IBS) is difficult to diagnose due to several factors [1,2]. Studies have found that four symptoms are the most common in IBS sufferers - bloating, pain relief with bowel movements, and loose and frequent bowel movements with the onset of pain [3,4]. A careful analysis can increase the diagnostic reliability (to positively diagnose IBS with 99% accuracy, the minimum score was 44 points) and reduce the examination effort for IBS. Kruis et al. found that a detailed history, physical examination, and basic laboratory tests are sufficient for a positive diagnosis of IBS [5]. Talley et al. concluded that the Manning criteria distinguish irritable bowel syndrome from organic gastrointestinal disease [6,7]. Soft and watery stool often served as an independent criterion. Jeong et al. and Boyce et al. concluded that the prevalence of IBS according to Manning, Rome I, and Rome II was 13.6%, 4.4%, and 6.9%, respectively [8,9,4]. Doğan and Unal showed that when the Manning criteria and the Kruis scoring system were applied together, these systems showed a strong correlation in IBS but not in esophagogastroduodenoscopy (OGD) [8,9]. Jellema et al. found that symptom-based IBS criteria cannot rule out organic disease and recommended that the Rome III criteria be reviewed in primary care [9,10]. This study evaluated the usefulness of the Manning, Kruis, and Rome IV criteria for IBS diagnosis using various standard definitions and attempted to determine the degree of agreement between these definitions. Repeated examinations should be avoided and the Bristol stool chart should be used to objectively describe bowel patterns and assign patients to the appropriate subtype [11].

## Materials and methods

Study design and setting

This retrospective observational cohort study was conducted from September 2019 to February 2020 on 130 IBS patients in the outpatient department of the Department of Surgery at the Vydehi Institute of Medical Sciences and Research Centre, Bangalore. In the first appointment, a thorough medical history was taken and a physical examination was performed on each patient. Prior to their diagnostic assessment, all patients who consented to participate completed the questionnaire. The questionnaire and scoring method for IBS was based on Manning criteria [6,7] and Kruis score [5]. The Rome IV criteria served as a comparison [2]. Investigations included complete blood count (CBC), erythrocyte sedimentation rate (ESR), C-reactive protein (CRP), and serum albumin. Statistical software SPSS 22.0 (Armonk, NY: IBM Corp.) and R environment version 3.2.2 (Indianapolis, IN: The R Foundation) were used for the analysis of the data. Additionally, Microsoft Word and Excel were used to create graphs, tables, etc.

Inclusion and exclusion criteria

Inclusion criteria included consenting patients over the age of 18 years who have had abdominal pain, bloating, and irregular bowel movements for more than six months. Exclusion criteria included patients with a history of organic gastrointestinal disease, patients on ventilator support due to acute respiratory distress syndrome (ARDS), patients with recurrent gastrointestinal infections, colorectal cancer, microscopic, lymphocytic, and collagenous colitis, celiac disease, inflammatory bowel disease (IBD), primary bile acid diarrhea, immune deficiency, and uncontrolled thyroid disease. Individuals with a history of liver disease or those whose liver enzyme levels were above normal were not included in the study. In addition, people who have undergone abdominal radiation or surgery other than appendectomy or cholecystectomy were excluded.

Statistical methods

This study performed a descriptive and inferential statistical analysis of Rome IV and compared it with Kruis and Manning tests. Results of continuous measurements are presented as mean standard deviation (min-max), and results of categorical measurements are presented in counts (%). Significance is assessed at a 5% level of significance. Assumptions were made about the data, e.g., that dependent variables should be normally distributed, samples drawn from the population should be random, and the cases of the samples should be independent. Student's t-test (two-tailed, independent) was used to find the significance of the study parameters on a continuous scale between two groups (intergroup analysis). Levene's test of homogeneity of variance was used to assess homogeneity. Chi-square/Fisher's exact test was used to determine the significance of study parameters on a categorical scale between two or more groups. Sensitivity, specificity, positive predictive value (PPV), negative predictive value (NPV), and accuracy levels were calculated to determine the diagnostic properties of Rome IV when compared to Kruis and Manning criteria. P-value=0.05<p<0.10 was considered suggestively significant, p-value 0.01<p≤0.05 was considered moderately significant, and p-value≤0.01 was considered strongly significant.

## Results

According to Table 1, the majority of patients (n=40, 30.8%) belonged to the age group of 41-50 years. This is followed by the second largest group of people (n=36, 27.7%) in the age group between 30 and 40 years. IBS is believed to be more common in people between the ages of 30 and 50 years, who make up more than 50% of the study participants.

**Table 1:** Gender distribution of the investigated patients.

Gender	No. of patients	Total (%)
Female	46	35.4
Male	84	64.6
Total	130	100.0

Table 2 shows that males make up 64.6% of the sample (n=84), while females make up 35.4% (n=46). When it comes to IBS subtypes, women are far more likely than men to have IBS with constipation. Gender differences and sex hormones may play a role in the pathogenesis of IBS.

**Table 2:** Age distribution of the study participants.

Age in years	No. of patients	Total (%)
<30	23	17.7
30-40	36	27.7
41-50	40	30.8
51-60	18	13.8
61-70	9	6.9
>70	4	3.1
Total	130	100.0

According to Table 3, the majority of patients under 30 years of age (n=101, 77.7%) had elevated ESR values. Forty-five patients (n=45, 34.6%) had CRP values greater than 1. CRP less than 1 was present in the remaining 65.4% of subjects. Four patients (n=4, 3.1%, >16 g/dL) had high hemoglobin levels. Eight patients (n=8) had elevated blood WBCs (>11×10^3^/µL). The Kruis score can be used to differentiate between organic bowel disease and IBS.

**Table 3:** Clinical variables of the Kruis score. CRP: C-reactive protein; ESR: erythrocyte sedimentation rate

Variables studied		No. of patients (n=130)	Total (%)
ESR (mm/h)	<30	101	77.7
	30-60	18	13.8
	>60	11	8.5
CRP (mg/dL)	<1	85	65.4
	>1	45	34.6
Hemoglobin (g/dL)	<12	17	13.1
	12–16	109	83.8
	>16	4	3.1
WBC (×10^3^/µL)	<7	65	50.0
	7-11	57	43.8
	>11	8	6.2

The reported dataset in Table 4 represents the sample population represented by the descriptive statistics of the coefficients (as determined by the Kruis score). ESR ranged from 1.00 to 120 with x̄=21.52 and SD=27.35 (mm/h) (normal=male 15 mm/h, female=20 mm/h, child=10 mm/h). C-reactive protein (CRP) ranged from 0.08 to 24.00 with x̄=1.12 and SD=2.37 mg/dL (normal=less than 0.3 mg/dL). Hemoglobin ranged from 7.60 to 43.20 with x̄=13.70 and SD=3.09 (g/dL). The white blood cell (WBC) count ranged from 3.00 to 17.70 with x̄=7.43 and SD=2.24 (per µL).

**Table 4:** Descriptive statistics (Kruis score). CRP: C-reactive protein; ESR: erythrocyte sedimentation rate

Variables	Min	Max	Mean	SD
ESR (mm/h)	1.00	120.00	21.52	27.35
CRP (mg/dL)	0.08	24.00	1.12	2.37
Hemoglobin (g/dL)	7.60	43.20	13.70	3.09
WBC (×10^3^/µL)	3.00	17.70	7.43	2.24

Table 5 shows that 56.2% (n=73) of patients were true positive and 43.8% (n=57) of patients were true negative. Likewise, for Manning criteria, 37.7% of patients (n=49) were true positive and 62.3% (n=81) were true negative. In the case of Rome IV criteria, 46.9% (n=61) of patients were true positive and 53.1% (n=69) of patients were true negative.

**Table 5:** Accuracy of the diagnostic tests - Kruis score, Manning criteria, and Rome IV criteria in irritable bowel syndrome.

Criteria		No. of patients (n=130)	Total %
Kruis	Negative	57	43.8
	Positive	73	56.2
Manning	Negative	49	37.7
	Positive	81	62.3
Rome IV	Negative	61	46.9
	Positive	69	53.1

Table 6 shows the results of 130 subjects who met the Rome IV diagnostic criteria; 61 subjects tested positive while 69 subjects were negative. The values were then compared for the same patient groups according to Kruis and Manning criteria. In the Kruis score, 62.3% (n=43) of the 69 patients who tested positive for the Rome IV criteria were true positive, and therefore, sensitive to both tests. Further, 50.8% of the patients (n=31) were true negative and thus specific for both tests. In this case, p=0.132 is not significant. For the Manning criteria, 63.8% (n=44) of the 69 patients who tested positive for the Rome IV criteria were true positive and thus sensitive to both tests. Additionally, 39.3% of the patients (n=24) were true negative and thus specific for both tests. Here, p=0.715 is not significant.

**Table 6:** Comparison of the Kruis score and Manning criteria with the Rome IV criteria for the assessment of IBS. P-values are calculated using the chi-squared (χ^2^) test and were considered not significant. IBS: irritable bowel syndrome

Criteria		Rome IV		Total (n=130)	p-Value
		Negative (n=61)	Positive (n=69)		
Kruis	Negative	31 (50.8%)	26 (37.7%)	57 (43.8%)	0.132
	Positive	30 (49.2%)	43 (62.3%)	73 (56.2%)	
Manning	Negative	24 (39.3%)	25 (36.2%)	49 (37.7%)	0.715
	Positive	37 (60.7%)	44 (63.8%)	81 (62.3%)	

Table 7 compares the Kruis and Manning IBS criteria with the Rome IV criteria. Based on the data presented above, the sensitivity and specificity of the Kruis score are 62.32 and 58.82, respectively. The sensitivity and specificity of Manning criteria are 63.77 and 39.34, respectively. The positive predictive value (PPV) and the negative predictive value (NPV) for Kruis are 58.90 and 54.39, respectively. The PPV and NPV values of the population criterion are 54.32 and 48.98, respectively. The accuracy of the Kruis score and the Manning criteria are 56.92 and 52.31, respectively. Our research led us to conclude that the Kruis score has superior specificity, PPV, and accuracy as compared to the Manning criteria.

**Table 7:** The sensitivity, specificity, and predictive value of the Kruis score with the Manning criteria for the assessment of IBS. PPV: positive predictive value; NPV: negative predictive value; IBS: irritable bowel syndrome

Criteria	Sensitivity	Specificity	PPV	NPV	Accuracy
Kruis	62.32	58.82	58.90	54.39	56.92
Manning	63.77	39.34	54.32	48.98	52.31

Table 8 compares the baseline clinical characteristics of patients using the Rome IV criteria. It was observed that 30.4% (n=21) of patients aged 41-50 years were true positive (sensitive), and 31.1% (n=19) of the patients in the age categories 30-40 years and 41-50 years were true negative (specific). In this case, p=0.729 is insignificant. The number of newly identified cases of a disease or condition per population at risk during a given period is commonly defined as incidence. Based on our data, with the total number of new cases being 21, a total risk population of 40, and a population size of 130 people, the incidence rate is 68.25 new cases per 130 people.

**Table 8:** A comparison of baseline clinical variables according to the Rome IV criteria of the participants.

Variables		Rome IV		Total (n=130)	p-Value
		Negative (n=61)	Positive (n=69)		
Age in years	<30	10 (16.4%)	13 (18.8%)	23 (17.7%)	0.729
	30-40	19 (31.1%)	17 (24.6%)	36 (27.7%)	
	41-50	19 (31.1%)	21 (30.4%)	40 (30.8%)	
	51-60	7 (11.5%)	11 (15.9%)	18 (13.8%)	
	61-70	3 (4.9%)	6 (8.7%)	9 (6.9%)	
	>70	3 (4.9%)	1 (1.4%)	4 (3.1%)	
Gender	Female	20 (32.8%)	26 (37.7%)	46 (35.4%)	0.560
	Male	41 (67.2%)	43 (62.3%)	84 (64.6%)	

Table 9 compares the clinical variables of patients assessed according to the Rome IV criteria, where the total (mean±SD) value of age was 42.69±13.52 years, ESR was 23.57±28.57 mm/h, CRP was 0.90±1.36 mg/dL, hemoglobin was 13.73±3.99 g/dL, and WBC was 7.15±2.28×10^3^/µL. As a result, the p-values for ESR, WBC, and CRP were not significant. As a result, the clinical variables listed above had no impact on the Rome IV criteria score.

**Table 9:** Clinical variables in participants screened according to the Rome IV criteria. CRP: C-reactive protein; ESR: erythrocyte sedimentation rate

Variables	Rome IV		Total	p-Value
	Negative	Positive		
Age in years	42.28±13.62	43.06±13.52	42.69±13.52	0.745
ESR (mm/h)	19.38±26.19	23.57±28.57	21.59±27.45	0.388
CRP (mg/dL)	1.37±3.15	0.90±1.36	1.12±2.38	0.263
Hemoglobin (g/dL)	13.69±1.66	13.73±3.99	13.71±3.10	0.947
WBC (×10^3^/µL)	7.74±2.19	7.15±2.28	7.43±2.25	0.136

## Discussion

IBS is a persistent and debilitating disease that affects between 9% and 23% of the world's population, with 12% seeking medical treatment in primary care [1]. Anand et al. found that 33% of IBS symptoms are caused in middle-aged men, with the prevalence increasing with age [11,12]. About a third of the patients see a doctor [11]. In our study, more than 22.3% of participants had an elevated ESR. CRP was elevated in 34.6%; 13.1% of participants had low hemoglobin levels, and it was observed that WBC increased by 6.2%. We found no anemia in the participants with IBS. Ford et al. found that single symptoms have limited accuracy in diagnosing IBS, and the Manning and Kruis scoring systems are only moderately accurate [12]. Despite widespread acceptance, only the Rome I categorization has been validated [13].

Whitehead and Drossman reviewed the evidence for the validity of symptom-based criteria for irritable bowel syndrome (Manning, Rome I, Rome II, and Rome III) [13,14]. Two types of validations have been reported - first, studies examining whether symptom criteria distinguish patients with structural disease from those without structural disease at colonoscopy, and second, whether symptom criteria distinguish individuals with IBS by positive diagnosis from healthy subjects or patients with other functional and structural disorders. Both types of research confirm the validity of the symptom-based IBS criteria. Rome III investigations are required. IBS has a significant impact on rural populations, with CRP levels being higher in IBS patients than in healthy controls [12]. However, they remain within normal laboratory values [15]. According to Halpin and Ford, symptoms consistent with IBS were significantly more common in patients with IBD than controls without IBD, even in those presumed to be in remission [16]. IBS-like symptoms were also much more common in Crohn's disease (CD) patients than in ulcerative colitis (UC) patients. Treatment options for IBD patients with IBS symptoms are critical.

Hauser et al. reported in a prospective study that IBS patients with higher ESR had a lower health-related quality of life (HRQoL), but there is no association between ESR and disease severity or overall HRQoL [17]. Lovell and Ford found in a meta-analysis that the prevalence of IBS varies across countries, with women being affected more often than men, but socioeconomic status has not been adequately studied [18]. Ford et al. found that more reliable IBS diagnostic approaches are needed to distinguish between IBS and organic diseases [19]. A systematic review and meta-analysis by Menees et al. to determine the efficacy of CRP, ESR, fecal calprotectin, and fecal lactoferrin in inflammatory bowel disease in patients with irritable bowel syndrome showed that CRP and calprotectin levels of 0.5 and 40 rule out IBD in IBS symptoms and improve diagnosis [20]. Oka et al. found that global IBS incidence varied significantly even when using the same diagnostic criteria and techniques. Rome IV criteria may be less relevant than Rome III criteria for population-based epidemiological surveys [21].

Limitations

First, the IBS patients from the included studies were defined using various criteria such as Manning criteria and Rome IV. Additionally, the IBS patients from the included studies are uncertain about the active phase or remission phase. Second, since the questionnaire represents the definition of IBS, the average score may be biased. Third, more studies are needed to determine if changing these criteria may improve accuracy. Fourth, a smaller sample yields a result that may not be sufficiently meaningful to detect a difference between groups, and the study may turn out to be a false negative indicating that a person does not have a specific disease.

## Conclusions

Existing diagnostic criteria do not distinguish IBS from organic disease, so a more detailed history and physical examination are needed to diagnose it. Laboratory markers should be used as a supplement to clinical observation and physical examination. In conclusion, our findings suggest that irritable bowel syndrome can be diagnosed using this scoring system, thereby addressing its numerous challenges. Further studies are needed to determine if changing these criteria can result in improved accuracy. We suggest that validation of the Rome IV criteria for population-based epidemiological surveys may be required. Currently used biomarkers are CRP, ESR, perinuclear antineutrophil cytoplasmic antibodies (pANCA), anti-*Saccharomyces cerevisiae *antibodies (ASCA), and fecal calprotectin; other biomarkers are yet to be confirmed in large clinical trials.

## References

[1] Saha L (2014). Irritable bowel syndrome: pathogenesis, diagnosis, treatment, and evidence-based medicine. World J Gastroenterol.

[2] Defrees DN, Bailey J (2017). Irritable bowel syndrome: epidemiology, pathophysiology, diagnosis, and treatment. Prim Care.

[3] Talley NJ, Phillips SF, Melton LJ (1990). Diagnostic value of the Manning criteria in irritable bowel syndrome. Gut.

[4] Boyce PM, Koloski NA, Talley NJ (2000). Irritable bowel syndrome according to varying diagnostic criteria: are the new Rome II criteria unnecessarily restrictive for research and practice?. Am J Gastroenterol.

[5] Kruis W, Thieme C, Weinzierl M, Schüssler P, Holl J, Paulus W (1984). A diagnostic score for the irritable bowel syndrome. Its value in the exclusion of organic disease. Gastroenterology.

[6] Manning AP, Thompson WG, Heaton KW, Morris AF (1978). Towards positive diagnosis of the irritable bowel. Br Med J.

[7] Talley NJ, Phillips SF, Melton LJ, Mulvihill C, Wiltgen C, Zinsmeister AR (1990). Diagnostic value of the Manning criteria in irritable bowel syndrome. Gut.

[8] Doğan UB, Unal S (1996). Kruis scoring system and Manning's criteria in diagnosis of irritable bowel syndrome: is it better to use combined?. Acta Gastroenterol Belg.

[9] Jellema P, van der Windt DA, Schellevis FG, van der Horst HE (2009). Systematic review: accuracy of symptom-based criteria for diagnosis of irritable bowel syndrome in primary care. Aliment Pharmacol Ther.

[10] Lacy BE, Patel NK (2017). Rome criteria and a diagnostic approach to irritable bowel syndrome. J Clin Med.

[11] Anand AC, Saiprasad GS, Bhalwar R (1998). A search for unhappy abdomen: prevalence of Irritable bowel syndrome in general population. Med J Armed Forces India.

[12] Ford AC, Talley NJ, Veldhuyzen van Zanten SJ, Vakil NB, Simel DL, Moayyedi P (2008). Will the history and physical examination help establish that irritable bowel syndrome is causing this patient's lower gastrointestinal tract symptoms?. JAMA.

[13] Whitehead WE, Drossman DA (2010). Validation of symptom-based diagnostic criteria for irritable bowel syndrome: a critical review. Am J Gastroenterol.

[14] Makharia GK, Verma AK, Amarchand R (2011). Prevalence of irritable bowel syndrome: a community based study from northern India. J Neurogastroenterol Motil.

[15] Hod K, Dickman R, Sperber A (2011). Assessment of high-sensitivity CRP as a marker of micro-inflammation in irritable bowel syndrome. Neurogastroenterol Motil.

[16] Halpin SJ, Ford AC (2012). Prevalence of symptoms meeting criteria for irritable bowel syndrome in inflammatory bowel disease: systematic review and meta-analysis. Am J Gastroenterol.

[17] Hauser G, Tkalcic M, Pletikosic S, Grabar N, Stimac D (2012). Erythrocyte sedimentation rate - possible role in determining the existence of the low grade inflammation in Irritable Bowel syndrome patients. Med Hypotheses.

[18] Lovell RM, Ford AC (2012). Global prevalence of and risk factors for irritable bowel syndrome: a meta-analysis. Clin Gastroenterol Hepatol.

[19] Ford AC, Bercik P, Morgan DG, Bolino C, Pintos-Sanchez MI, Moayyedi P (2013). Validation of the Rome III criteria for the diagnosis of irritable bowel syndrome in secondary care. Gastroenterology.

[20] Menees SB, Powell C, Kurlander J, Goel A, Chey WD (2015). A meta-analysis of the utility of C-reactive protein, erythrocyte sedimentation rate, fecal calprotectin, and fecal lactoferrin to exclude inflammatory bowel disease in adults with IBS. Am J Gastroenterol.

[21] Oka P, Parr H, Barberio B, Black CJ, Savarino EV, Ford AC (2020). Global prevalence of irritable bowel syndrome according to Rome III or IV criteria: a systematic review and meta-analysis. Lancet Gastroenterol Hepatol.

